# Concurrent exergaming and transcranial direct current stimulation to improve balance in people with Parkinson’s disease: study protocol for a randomised controlled trial

**DOI:** 10.1186/s13063-018-2773-6

**Published:** 2018-07-16

**Authors:** Dale M. Harris, Timo Rantalainen, Makii Muthalib, Liam Johnson, Rachel L. Duckham, Stuart T. Smith, Robin M. Daly, Wei-Peng Teo

**Affiliations:** 1 Institute for Physical Activity and Nutrition (IPAN), School of Exercise and Nutrition Sciences, Deakin University, Geelong, VIC Australia; 2 SilverLine Research Services, Brisbane, QLD Australia; 3 Clinical Exercise Science Research Program, Institute of Sport Exercise and Active Living (ISEAL), Victoria University, Melbourne, VIC Australia; 4 The Florey Institute of Neuroscience and Mental Health, University of Melbourne, Melbourne, VIC Australia; 5 Australian Institute for Musculoskeletal Sciences (AIMSS), St. Albans, VIC Australia; 6 Southern Cross University, Coffs Harbour, NSW Australia

**Keywords:** Balance, Exergaming, Cognition, TMS, fNIRS

## Abstract

**Background:**

People with Parkinson’s disease (PD) commonly experience postural instability, resulting in poor balance and an increased risk of falls. Exercise-based video gaming (exergaming) is a form of physical training that is delivered through virtual reality technology to facilitate motor learning and is efficacious in improving balance in aged populations. In addition, studies have shown that anodal transcranial direct current stimulation (a-tDCS), when applied to the primary motor cortex, can augment motor learning when combined with physical training. However, no studies have investigated the combined effects of exergaming and tDCS on balance in people with PD.

**Methods/design:**

Twenty-four people with mild to moderate PD (Hoehn and Yahr scale score 2–4) will be randomly allocated to receive one of three interventions: (1) exergaming + a-tDCS, (2) exergaming + sham a-tDCS or (3) usual care. Participants in each exergaming group will perform two training sessions per week for 12 weeks. Each exergaming session will consist of a series of static and dynamic balance exercises using a rehabilitation-specific software programme (Jintronix) and 20 minutes of either sham or real a-tDCS (2 mA) delivered concurrently. Participants allocated to usual care will be asked to maintain their normal daily physical activities. All outcome measures will be assessed at baseline and at 6 weeks (mid-intervention), 12 weeks (post-intervention) and 24 weeks (3-month follow-up) after baseline. The primary outcome measure will be the Limits of Stability Test. Secondary outcomes will include measures of static balance, leg strength, functional capacity, cognitive task-related cortical activation, corticospinal excitability and inhibition, and cognitive inhibition.

**Discussion:**

This will be the first trial to target balance in people with PD with combined exergaming and a-tDCS. We hypothesise that improvements in balance, functional and neurophysiological outcome measures, and neurocognitive outcome measures will be greater and longer-lasting following concurrent exergaming and a-tDCS than in those receiving sham tDCS or usual care.

**Trial registration:**

Australian New Zealand Clinical Trials Registry, ACTRN12616000594426). Registered on 9 May 2016.

**Electronic supplementary material:**

The online version of this article (10.1186/s13063-018-2773-6) contains supplementary material, which is available to authorized users.

## Background

Parkinson’s disease (PD) is a chronic neurodegenerative disorder that affects approximately 1% of adults aged 60 years and older worldwide [1–3]. A prominent motor symptom of PD is postural instability, which typically worsens as the disease progresses, leading to impaired balance and increased risk of falls [4, 5]. The percentage of people with PD who experience a fall ranges from 46% to 55% [6–8], with falls contributing to injury, loss of independence and reduced quality of life (QoL) [5, 8–11]. Exercise is an effective treatment for improving balance in people with PD [4–7], and ongoing exercise training can be achieved through home-based training [8].

Exercise-based video gaming (exergaming) and immersive virtual reality modes have been used as part of home-based rehabilitation programmes to improve balance [9], and they may be as effective as conventional balance training in people with PD [10]. Exergaming combines physical movement with motion capture technology (e.g., Kinect®, Microsoft, Redmond, WA, USA; Wii®, Nintendo, Kyoto, Japan) to generate an on-screen avatar, which mimics the participant’s movements in real time. This is known as gesture-based interaction training [11], which combines automated game instructions as well as auditory and haptic inputs to correct performance and sustain motivation levels during and following game play [12–14]. For example, exergames for rehabilitation can provide auditory instructions to improve patient performance, such as ‘stand tall’ or ‘place your feet wider’, allowing patients to recognise deficits in movement and self-correct in real time [10, 15]. In addition, given that people with PD are dependent on sensory cues to overcome postural instability [16, 17], exergames employ visual and auditory feedback (termed *biofeedback*) techniques to create a quasi-immersive environment which can facilitate motor and cognitive learning [12, 18–22].

People with PD have difficulties with the long-term consolidation of new motor skills [23], but adjunctive neuromodulatory techniques, such as non-invasive brain stimulation, may help to upregulate neuroplasticity and facilitate motor skill acquisition and retention [24]. Transcranial direct current stimulation (tDCS) is a therapeutic tool that has been used to attenuate motor symptoms of PD [24, 25] with few side effects (e.g., occasional headache/nausea and scalp itchiness) [24]. When applied to the primary motor cortex (M1), anodal tDCS (a-tDCS) can induce a lasting increase in M1 excitability and reduce cortical inhibition, resulting in improved functional performance [26]. When combined with other training modalities such as exercise or physical therapy, greater and longer-lasting improvements in motor function have been observed in healthy and clinical populations than with either treatment modality alone [27–29]. However, no studies have investigated the concurrent use of a-tDCS and exergaming in people with PD.

The aims of this study are to determine the effects of a 12-week concurrent exergaming and a-tDCS intervention on measures of static and dynamic balance and to evaluate any long-term residual effects. Additionally, we will employ transcranial magnetic stimulation (TMS) techniques in the M1 and functional near-infrared spectroscopy (fNIRS) in the left and right dorsolateral pre-frontal cortices (DLPFC) to determine neurophysiological mechanisms that may underpin changes in balance. We hypothesise that the combination of exergaming and a-tDCS will lead to greater and longer-lasting improvements in balance and neurophysiological measures than exergaming or usual care alone.

## Methods/design

We will conduct a 24-week, exploratory, double-blind, randomised controlled trial to investigate the effects of a concurrent exergaming and a-tDCS intervention that includes a 12-week post-intervention follow-up period. Participants will be randomly allocated to one of three groups: (1) exergaming + a-tDCS, (2) exergaming + sham a-tDCS or (3) usual care (control). The trial will be conducted at the Institute for Physical Activity and Nutrition at Deakin University, Burwood, Melbourne, Australia. Ethical approval was granted by the Deakin University Human Research Ethics Committee (project number 2016-053), and the trial is registered with the Australian New Zealand Clinical Trials Registry (ACTRN12616000594426).

### Participants

A total of 24 men and women aged 55 years and older who have been diagnosed with mild to moderate PD (Hoehn and Yahr scale scores 2–4) will be invited to participate in this study.

### Recruitment

A list of people with PD will be compiled from an existing research study database at Deakin University and invited by telephone to participate in this trial. PD support groups within inner and outer suburban Melbourne will also be approached via mail or telephone to attend a study information seminar, which will be delivered by a member of the research team. All interested support group members will receive additional information pamphlets. Study advertisements will also be placed in the Australian Parkinson’s Disease Registry, in local newspapers and on noticeboards at community libraries. All participants who express an interest in the study will first be screened over the telephone, and eligible participants will then be required to come to Deakin University for additional screening as outlined below.

### Screening and eligibility

#### Telephone screening

All potential participants will be screened first over the telephone and will be eligible for this trial if they meet the following criteria: (1) have been diagnosed with PD by a neurologist, (2) have experienced one or more falls in the last 12 months, and (3) are not performing any regular physical therapy or structured exercise training. Participants will be ineligible based on the following criteria: (1) severe lower limb motor impairments and/or requirement of a walking aid or wheelchair, (2) previously diagnosed with stroke or dementia, (3) having metal implants in the head (i.e., deep brain stimulator or aneurysm clips), or (4) any other known medical, mental health or physical condition which may interfere with balance. Last, all participants need to be on a stable medication regimen for more than 6 weeks before proceeding to the on-site screening session.

#### On-site screening

The on-site screening session will be held at Deakin University, Burwood campus. During this session, participants will be required to complete the Montreal Cognitive Assessment, which is a 30-point screening instrument that assesses mild cognitive impairment (MCI). A cut-off score < 26 of 30 indicates the presence of MCI and will exclude participants from this trial [16, 30]. Participants will also be assessed using the motor section of the Unified Parkinson’s Disease Rating Scale (UPDRS), and individuals will be deemed eligible for the study only if they have mild to moderate PD (Hoehn and Yahr scale scores 2–4). All eligible participants will then be given a plain-language statement and consent form to sign and return prior to commencing the programme.

### Randomisation

**Fig. 1:**
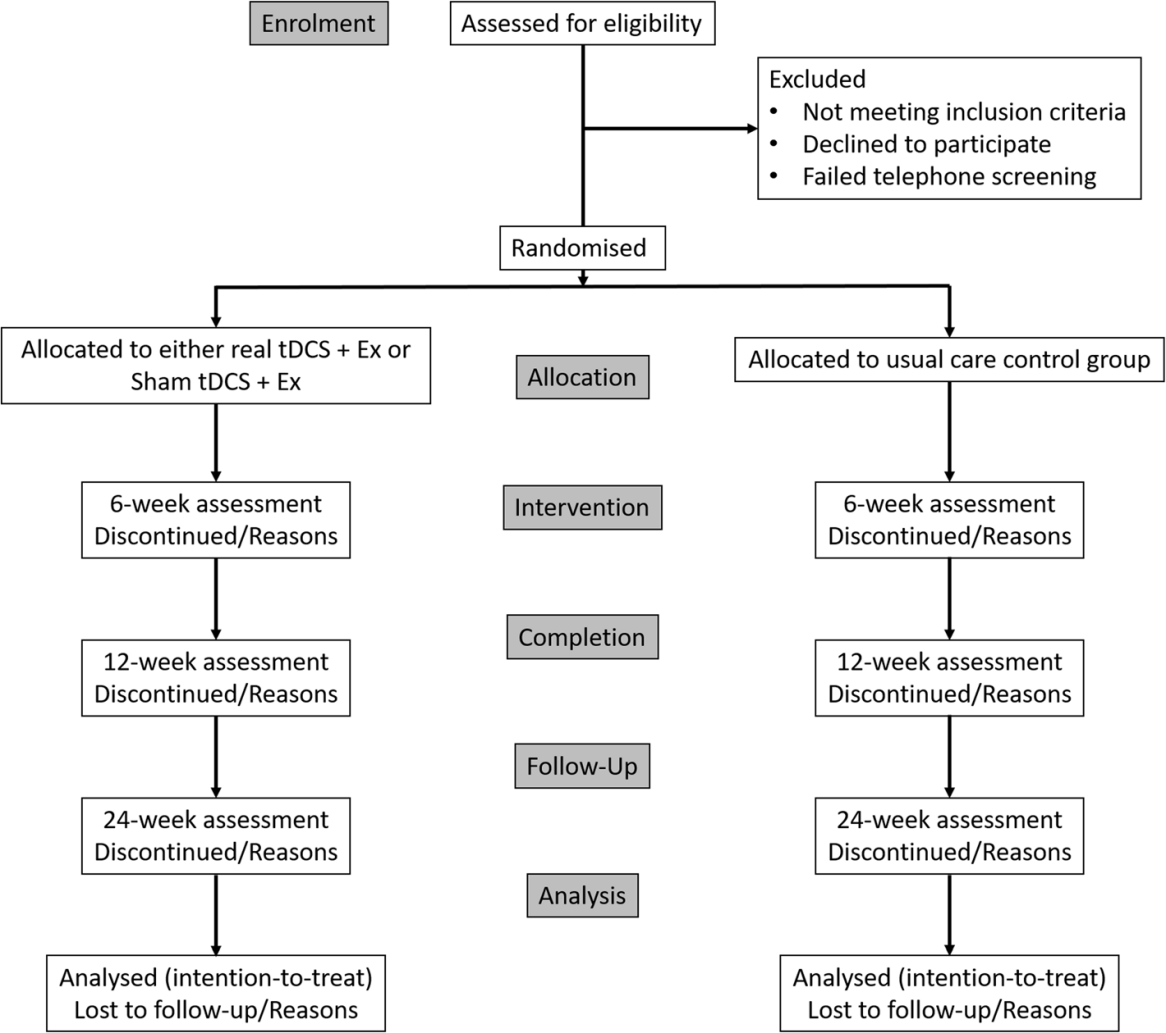
Consolidated Standards of Reporting Trials (CONSORT) flow diagram of eligibility screening to the final follow-up assessment. *tDCS* Transcranial direct current stimulation

Randomisation will be performed by a researcher independent from the study using computer-generated random numbers (Excel software; Microsoft). A flow diagram of the study protocol is provided in Fig. 1.

### Familiarisation

Following enrolment in the study and randomisation, participants will be asked to attend a familiarisation session at Deakin University. During this session participants allocated to the intervention groups will briefly practise all testing and intervention procedures. Participants allocated to the control group will only briefly undertake the testing procedures. As part of the intervention familiarisation procedure, all participants will perform a single set of each exergame while the objectives of each exergame are explained to them.

### Allocation and blinding

Following their enrolment in the study and after baseline testing, participants will be randomly allocated to one of three groups: (1) exergaming + a-tDCS, (2) exergaming + sham tDCS group or (3) a usual care control group. All groups will be stratified by sex to ensure equal distribution of male and female participants between groups. A researcher independent from the research team will pre-allocate a code for either real or sham a-tDCS, and the code will be given to the attending researcher to be input into the tDCS device during each training session. The identity of each code will be known only by the independent researcher to ensure double-blinding of both the attending researcher conducting the training session and the participant.

### Intervention

**Fig. 2:**
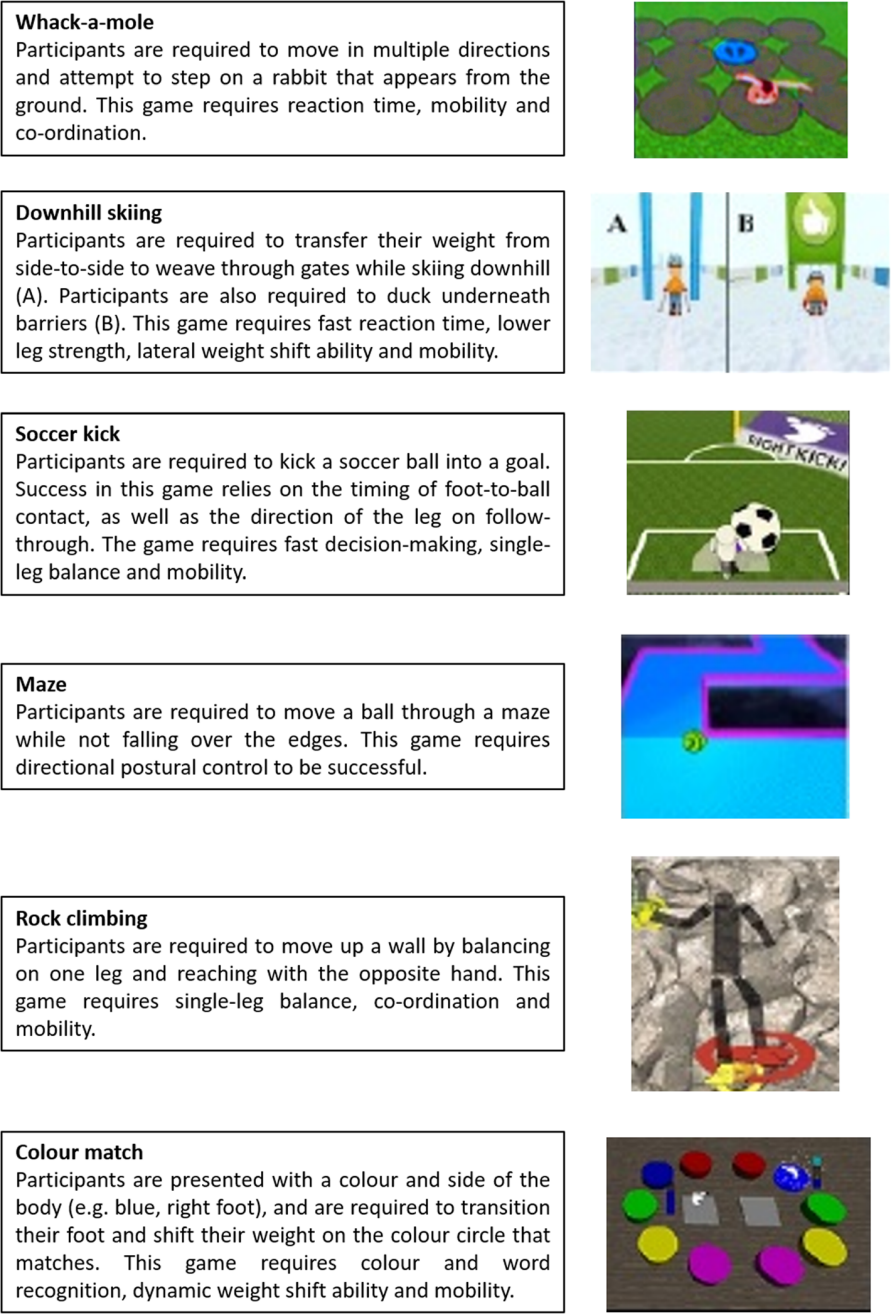
Example exergaming programme to improve balance

The training intervention will be conducted over a 12-week period and will consist of exergames using an augmentative virtual reality software (Jintronix, Montreal, QC, Canada) with concurrent a-tDCS. Participants will be required to attend training sessions at Deakin University, Burwood campus, twice per week on non-consecutive days (24 training sessions in total). The exergaming training sessions will be supervised by the attending researcher and will last approximately 30 minutes. Jintronix rehabilitation software, which contains over 100 rehabilitation exergames, along with a three-dimensional motion capture sensor (Kinect®), will be used in this study. The exergames performed by the participants will be pre-selected by the attending researcher and tailored to each participant’s physical needs. The participant’s programmes will include exercises that focus on multiple domains of balance, including bilateral weight transfer, unilateral balance ability, trunk mobility and transitional activities (e.g., standing from a chair). An example balance training programme is illustrated in Fig. 2.

**Fig. 3:**
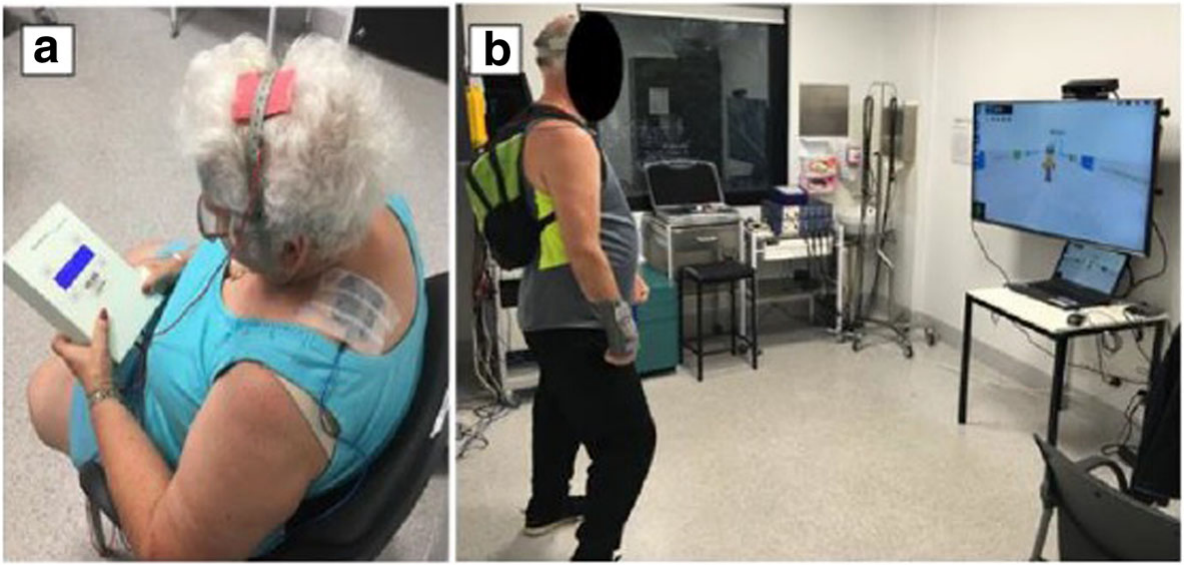
Example of anodal transcranial direct current stimulation montage (**a**) and exergaming set-up using Jintronix software (**b**)

The exergames will be projected on a wall-mounted flat screen television via a laptop (Fig. 3). The attending researcher will provide standardised verbal feedback to encourage performance throughout each exergaming session. As an embedded feature of each exergame, a completion score will be provided to the participant on-screen, which will represent their performance; for example, a score of five of ten gates achieved in the downhill ski exergame indicates a 50% success rate. The training sessions will be progressed manually by the attending researcher by increasing the difficulty of each exergame. Gaming progression will be based on the participant’s capacity to consistently achieve a score of 80% over two consecutive sessions.

A tDCS Stimulator Model 101 (TCT Research Ltd., Kowloon, Hong Kong, China) will be used to deliver real and sham a-tDCS concurrently during exergaming using rubber electrodes (50 mm × 70 mm) placed on the scalp over the M1 to the area which best represents the lower limb (Fig. 3). The optimal lower limb motor representation will be pre-determined by TMS during baseline testing. A midline monopolar anode-electrode montage will be adopted [31], with the cathode placed on the trapezius muscle at the midpoint between the occipital protuberance and acromion process/lateral portion of the scapula spine. For a-tDCS, the intensity will be incrementally increased over the initial 30 seconds to 2 mA and then maintained at that intensity for the duration of the training. Sham a-tDCS will include a 30-second ramp phase to 2 mA to provide sensory stimulation similar to that of real anodal tDCS before returning to baseline. This method of sham stimulation has been used successfully in a-tDCS to blind the participant and attending researcher to the stimulation condition [25, 32]. To ensure double-blinding of the participant and experimenter administering tDCS, blinding codes will be provided to the experimenter prior to the start of the session that activates either the sham or active a-tDCS option.

### Usual care

Participants assigned to the control group will be instructed to continue to receive their usual care from their medical practitioner and community services. In addition, usual care participants will be asked to refrain from partaking in any physical exercise training for the duration of the intervention (i.e., 24 weeks from baseline testing) other than their usual activities of daily living. Upon completion of the follow-up assessment, usual care participants will be offered advice about exercises from the attending researcher.

### Outcome measures

**Fig. 4:**
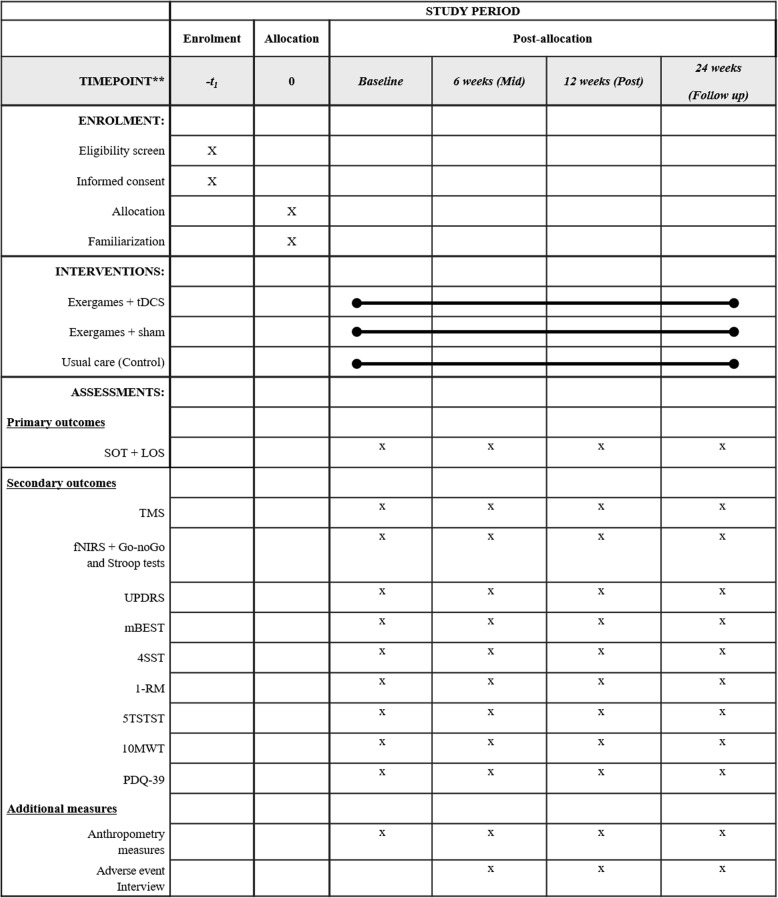
Standard Protocol Items: Recommendations for Interventional Trials (SPIRIT 2013) diagram illustrating the schedule of enrolment, post-allocation and close-out for all assessments. *TMS* Transcranial magnetic stimulation, *fNIRS* Functional near-infrared spectroscopy, *UPDRS* Unified Parkinson’s Disease Rating Scale, *mBEST* Mini Balance Evaluation Systems Test, *4SST* Four Square Step Test, *5TSTST* Five Times Sit to Stand Test, *10MWT* 10-Metre Walk Test, *PDQ-39* 39-Item Parkinson’s Disease Questionnaire

A summary of all outcome measures collected at each time point is shown in Fig. 4 (*also see* Additional file 1: SPIRIT checklist). All outcome measures will be taken at four time points: baseline (week 0), mid-intervention (6 weeks), post-intervention (12 weeks) and follow-up (24 weeks). The mid- and post-intervention testing sessions will occur within 72 hours, but no less than 24 hours, following the preceding training session.

### Primary outcome measure

#### Dynamic posturography

The primary outcome will be dynamic balance, which will be measured using the Limits of Stability Test (LOS) delivered through a dynamic computerised posturographic device (NeuroCom SMART Balance Master; Natus Medical, Inc., Pleasanton, CA, USA). The NeuroCom SMART Balance Master consists of two movable force platforms mounted with five strain gauges (100-Hz sampling rate) sensitive to vertical and horizontal shear forces and a visual surround designed to prevent peripheral visual fixation [33]. The force plate and visual surroundings are designed to tilt in an anterior-posterior plane during various testing conditions. A telemonitor mounted on the visual surround provides instructions for each test in addition to visual feedback of the participant’s foot position and movement during the test [33]. The participants will be asked to stand barefoot on the centre of the force platform. Participants will be fitted into a support harness throughout the test to prevent a fall occurring. The two posturographic tests to be performed on the NeuroCom SMART Balance Master are the LOS and the Sensory Organization Test (SOT), which is a secondary outcome measure. The LOS requires each participant to purposefully move their centre of pressure (COP) towards an intended target in the following order: forward, forward right, right, backward right, backward, backward left, left and forward left [27]. Movement velocity (degrees per second), maximal excursion (greatest distance [cm] achieved towards the intended target/maximal distance of target [cm]) and directional control ([the amount of intended movement − amount of extraneous movement)/amount of intended movement] × 100) will be measured. The LOS has shown excellent test-retest reliability for movement velocity (intraclass correlation coefficient [ICC] = 0.825) and sway path length (0.846) [34].

### Secondary outcome measures

#### Static posturography

The SOT comprises six different sensory conditions in quiet stance and measures the COP path in centimetres. The test begins with condition 1 (eyes open in a quiet stance) and sequentially moves through each condition, which progressively becomes more challenging by either distracting or removing visual and/or proprioceptive feedback. The visual surround and force platform are sway-referenced, which refers to the tilting of the support surface and/or visual surround in response to movement of the participant’s COP. During the sway-referenced conditions, erroneous sensory information is presented to discombobulate the participant while the force platform measures the participant’s ability to compensate by using other senses to maintain balance equilibrium. The following are the six sensory conditions:

1. *Condition 1*: Eyes open and fixed support
2. *Condition 2*: Eyes closed and fixed support
3. *Condition 3*: Eyes open sway-referenced and support sway-referenced
4. *Condition 4*: Eyes open and support sway referenced
5. *Condition 5*: Eyes closed and support sway-referenced
6. *Condition 6*: Eyes open and sway-referenced with support sway-referenced

The composite equilibrium score, presented as a weighted average percentage of the six conditions, will be used to determine overall static balance performance and has shown good reliability (ICC = 0.67) [35]. Additionally, the sensory analysis ratios obtained from specific sensory test condition pairs will be used to determine the level of reliance on different senses to maintain balance equilibrium [36]. The following ratios will be used:

- Somatosensory ratio ([SOT2/SOT1] × 100) will measure the participant’s reliance on the somatosensory system to maintain balance equilibrium.
- Visual ratio ([SOT4/SOT1] × 100) will measure the participant’s reliance on the visual system to maintain balance equilibrium.
- Vestibular ratio ([SOT5/SOT1] × 100) will measure the participant’s reliance on the vestibular system to maintain balance equilibrium.
- Visual preference ratio ([SOT 3 + 6/SOT 2 + 5] × 100) will measure the participant’s reliance on visual information, even when the information is incorrect, to maintain balance equilibrium.

Participants will have only a single practise trial for each sensory condition to minimise any practise effect between testing protocols. If the participant requires assistance or takes a considerable step away from the force plate, it will be registered as a loss of balance, and an equilibrium score of zero will be indicated.

#### Transcranial magnetic stimulation and electromyography

Single- and paired-pulse TMS of the M1 will be conducted using a BiStim^2^ 200^2^ magnetic stimulator (Magstim Co., Whitland, UK) to measure changes in corticospinal excitability and intracortical inhibition of the tibialis anterior (TA) muscle. The TA was selected as the target muscle because it is primarily involved with postural balance [37]. Prior to TMS, all participants will wear a custom-made nylon cap marked with a 1-cm concentric grid that extends outward from the vertex. A double-cone TMS coil with a wingspan of 110 mm will be positioned directly over the vertex of the head, and the optimal site of stimulation will be determined by initial exploration around the vertex that produces the largest and most consistent motor-evoked potential (MEP) recordings from the TA. Once the optimal site of stimulation has been identified, the resting motor threshold (RMT) will be determined. The RMT is defined as the lowest TMS intensity that produces an average MEP amplitude between 50 and 100 μV in response to five of ten stimuli [38]. Ten single-pulse TMS stimuli will then be delivered at 80%, 100%, 120%, 140% and 160% of RMT each to establish a stimulus-response curve that will be used to measure changes in corticospinal excitability with excellent reliability (ICC = 0.986) [38].

Corticocortical inhibitory circuits are suppressed in people with PD [39], and therefore paired-pulse TMS will be used to determine changes in short-interval/long-interval intracortical inhibition (SICI/LICI). Paired-pulse TMS will be delivered using a conditioning stimulus-test stimulus paradigm separated by a set interstimulus interval (ISI), which has shown excellent reliability (ICC = 0.934) [38]. To measure SICI, the CS will be set at 0.8 RMT, whereas the TS will be set at 1.2 RMT, separated by an ISI of 3 milliseconds [40]. For LICI, both the CS and TS will be set at 1.2 RMT, separated by an ISI of 100 milliseconds [41]. To calculate SICI and LICI, ten conditioned and unconditioned TS at the respective ISIs will be delivered and expressed as a percentage change of the average conditioned to unconditioned TS response. Surface electromyography (sEMG) can detect the level of muscle activity induced from transcranial stimulation and will be recorded using two bipolar Ag-AgCl electrodes 2 cm apart over the muscle belly of the TA and a ground electrode placed over the patella [42]. Prior to applying the electrodes, the participant’s skin will be shaved and swabbed with alcohol. All sEMG signals will be amplified (× 1000) with band-pass filtering between 5 Hz and 500 Hz, sampled at 4000 Hz and collected via a laboratory analog-digital interface (PowerLab 8/30; ADinstruments, Bella Vista, Australia) for later offline analysis.

#### Cognitive function

E-Prime software (Psychology Software Tools, Sharpsburg, PA, USA) [43] will be used to design two fNIRS-compatible computer-based cognitive assessments using a three-block design with a 30-second task period offset with a 30-second rest period. The Stroop colour-word test and the Go/No-Go test will be used to assess executive function, in particular response inhibition, interference control, attention and choice reaction time. In the Stroop colour-word test, participants will be presented with two conditions: incongruent and congruent. The incongruent condition will randomly present two colour words (red and green) written in opposing ink colours (e.g., the word ‘red’ will be written in green ink). For the congruent condition, the word and ink colour are the same (e.g., the word ‘red’ will be written in red ink). Upon presentation of the word, the participant must strike the key indicating the colour of the word presented, and the response time will be recorded in seconds. Cognitive central processing ability will be determined by measuring the difference in response times between the two conditions [29].

The Go/No-Go task is a choice reaction time test that requires participants to either strike or delay striking the left mouse button during two separate conditions (go and no-go). In the ‘go’ condition, the participants will be presented with a random series of letters and will be asked to strike the left mouse button as fast as they can as each letter appears. In the ‘no-go’ condition, a further random series of letters will appear; however, the participant will be asked to delay striking the mouse only when the letter ‘X’ appears. The reaction time for each response during each condition will be recorded in seconds.

#### Functional near-infrared spectroscopy

fNIRS is a non-invasive and portable neuroimaging technique that uses near-infrared light which penetrates the skull to detect changes in oxygenated haemoglobin (O_2_Hb) and deoxygenated haemoglobin (HHb) concentrations within the cortical microcirculation, such as the capillary, arteriolar and venular beds. fNIRS will be used in conjunction with the Stroop and Go/No-Go tasks to measure task-related changes in DLPFC activation. Task-related changes in fNIRS haemodynamic response activation show adequate to high reliability (ICC = 0.42–0.87) [44] and have been validated against the gold standard functional magnetic resonance imaging (fMRI) blood oxygen level-dependent response [45, 46].

A multi-channel continuous-wave fNIRS system (OxyMon Mk III; Artinis Medical Systems, Elst, The Netherlands) will be used to measure task-related changes in O_2_Hb and HHb concentrations in the left and right DLPFC during the Stroop and Go/No-Go tasks [47, 48]. An eight-channel fNIRS optode montage will be used (four channels on each hemisphere), and each transmitter and receiver optode pair will be placed between 30 mm and 40 mm apart for optimal near-infrared light penetration into the cortical layer.

Oxysoft data acquisition software (Artinis Medical Systems) will be used for fNIRS data collection and analysis. The modified Lambert-Beer law, assuming constant scattering [49], will be adopted to detect concentration changes of O_2_Hb and HHb in the left and right DLPFC, dependent on the changes in detected light intensity. For analysis, O_2_Hb and HHb signals of the four channels of each hemisphere will be averaged. The moving SD based artefact removal method will be used within each trial to remove movement artefacts and other noise from the fNIRS signals [50]. The thresholds for artefact detection will be set to 0.45 for O_2_Hb and 0.18 for HHb [51], with a window length for moving SD calculation at 0.5 seconds, and a window length for artefact correction (LOESS smoothing window) at 1 second. The fNIRS signals will then be linearly de-trended per trial and low-pass-filtered at 0.1 Hz to remove heart rate and other high-frequency physiological signals. To enable direct comparison of the different trials within each task, the filtered signals will be biased using the average concentration of the 5 seconds before the “start” instruction as a reference (zero). Then, individual trials will be averaged per task to create three mean time-course signals per person, which will then be averaged over all participants. Last, the peak and mean concentrations of O_2_Hb and HHb will be calculated over all trials for all participants and then averaged for each of the tasks.

#### Quality of life

The 39-item Parkinson’s Disease Questionnaire (PDQ-39) is a valid and reproducible PD-specific measure of QoL [52, 53]. Each question is based on a 5-point ordinal scoring system ranging from 0 = never to 4 = always. There are eight dimensions, with each dimension score being the sum score of each item in the dimension divided by the maximum possible score of all the items in the dimension, multiplied by 100. The overall score is known as the Parkinson’s disease summary index and is the sum of all dimension total scores divided by 8.

#### Functional assessments

The UPDRS, Mini Balance Evaluation Systems Test (mBEST), Five Times Sit to Stand Test (5TSTST), 10-Metre Walk Test (10MWT), Four Square Step Test (4SST) and maximal lower limb strength (one-repetition maximum [1RM] leg press) will be used to assess disease-related motor function and lower extremity physical function. Each of these tests will be performed in the same order each time with the participants wearing their own footwear and will be conducted by the attending researcher.

Section III of the UPDRS will be used to measure disease-related motor function and has shown excellent reliability (ICC = 0.90) [54]. The UPDRS comprises 33 scores based on 18 questions with several right, left or other body distribution scores.

The mBEST is a reliable tool (ICC = 0.92) [55] that assesses four domains of dynamic balance, including anticipatory postural adjustments, reactive postural control, sensory orientation and dynamic gait. The testing series consists of 14 tests, with each of the 14 items rated on a 3-level ordinal scale (ranging from 0 = cannot perform/severe, to 2 = normal) for a total score of 28 points. For the two items that are assessed bilaterally, the lower score will be used for the composite score. A cut-off score of 20 of 28 will be used to distinguish participants as ‘at risk’ for falls [55].

The 5TSTST will be used to determine functional lower limb power and has excellent test-retest reliability for people with PD (ICC = 0.91) [56]. Participants begin the 5TSTST test sitting in an armless chair with a height of 45 cm [34]. The participants will be instructed to cross their arms over their chest and sit with their back against the upright back-rest of the chair, with their feet placed flat on the floor and their knees at 90 degrees. They will then be instructed to quickly and repeatedly rise to a full standing position and then sit back down on the chair for five repetitions. A single repetition will be defined as a vertical standing position with an upright trunk and with the hips and knees extended. The test will begin once the participant moves off the chair for the first stand and will finish once the participant sits following the fifth sit-to-stand repetition, with the total time to completion recorded in seconds.

The 10MWT will be used to determine gait speed and has shown excellent test-retest reliability for comfortable gait speed (ICC = 0.96) and maximum gait speed (ICC = 0.97) for people with PD [57]. The test is set up with two cones positioned 10 m apart, with two more cones positioned at the 2-m and 8-m marks. The participant is instructed to walk the entire 10 m, but only the time from the moment the participant reaches the 2-m cone to the moment they reach the 8-m cone (distance of 6 m) is recorded to accommodate for acceleration and deceleration periods. Each participant will perform three trials at two different walking speeds: comfortable speed and fast speed. Each trial will be separated by a 1-minute rest period, and each trial will be recorded in seconds. The average speed in metres per second will be calculated and used for analysis [57].

The 4SST will be used to determine dynamic balance and stepping speed in four directions [58]. The 4SST has shown excellent test-retest reliability (ICC = 0.98) for people with PD [59]. Participants will be asked to step forwards, sideways and backwards over two metal rods positioned flat on the floor in a cross formation. The test begins with the participant moving first in a clockwise direction and returning in a counter-clockwise position to the start square. Participants will be asked to step with two feet into each square without touching or stepping on the rods as quickly as possible. A practise trial will be performed first, followed by two true attempts, which will be recorded with a stopwatch. The fastest time of completing the sequence in seconds will be used for analysis.

#### Functional strength of the lower limb

Lower extremity strength will be assessed using a 1RM leg press, which has previously shown excellent test-retest reliability for people with PD (ICC = 0.87–0.98) [60] and will be conducted using fixed pneumatic leg press (Air300; KEISER, Fresno, CA, USA). Ten warm-up repetitions will be performed prior to any near-maximal effort. The participant will then perform a near-maximal repetition (estimated at 80% of their 1RM) while maintaining correct technique, following which the resistance will be incrementally increased by 5–10% as appropriate, until the participant can no longer perform a full repetition [61]. Three minutes’ rest will separate each maximal attempt, and verbal encouragement will be provided. The highest resistance used to perform a successful repetition will be recorded as the 1RM in kilogrammes.

### Additional measures

Height will be measured to the nearest 0.1 cm using a standardised portable stadiometer. Weight will be measured to the nearest 0.1 kg using a standardised set of scales. Both height and weight will then be used to calculate body mass index in kilogrammes per metre squared. To ensure consistency of the data collection, participants will be instructed to wear light-fitting clothes and remove loose items before standing barefoot while having their height and weight recorded. Finally, interviews will be conducted with participants to monitor habitual physical activity and to establish any medication (in addition to PD-related medication) that they may be taking throughout the length of the intervention.

### Exercise compliance

Participants will be required to complete a minimum of 20 of the 24 scheduled training sessions (representing > 85% attendance) within the entirety of the 12-week intervention period. If sessions are missed, participants will be given an opportunity to complete additional sessions to make up for the missed sessions. This completion percentage is typical of large community-based exercise intervention studies and meets standard expectations of per-protocol analysis [62]. Compliance will be recorded using attendance sheets, which will be filled out by the attending researcher after each training session.

### Adverse events

The attending researcher will discuss what an adverse event (AE) is with each participant before the intervention begins. For this trial, an AE will be defined as any unfavourable and unintended sign, symptom or disease temporarily associated with the intervention (either exergaming or a-tDCS), without any understanding about causality or relationship to the therapy. The attending researcher will ask each participant before training begins about any AEs experienced and will document all AEs associated with the intervention. Usual care participants will be phoned and asked fortnightly about any experienced AEs. All AEs will be reported following the guidelines recommended by the National Health and Medical Research Council position statement (https://www.nhmrc.gov.au/guidelines-publications/e112). All participants will be asked to contact the attending researcher immediately after experiencing an AE, which will be recorded and promptly reported to the chief investigator, who will then determine its seriousness and causality in conjunction with any medical staff attending the event. Any AEs that are related to any part of the intervention will be reported to the Deakin University Human Research Ethics Committee.

### Sample size calculation

On the basis of data provided by Shih et al. [31], whose primary outcome was the LOS to assess reaction time and excursion endpoints in people with PD (Cohen’s *d* = 0.74, 77% improvement in reaction time, 89% improvement in endpoint excursion), a sample size calculation using G*Power statistical software estimated a sample size of five participants per group would be sufficient for 85% power (α = 0.05) while using a conservative estimated SD change of ± 5. It is difficult to determine if this is a large-enough sample size to detect change between the real and sham a-tDCS conditions, given the paucity of literature using a testing paradigm of exercise + real a-tDCS vs exercise + sham a-tDCS vs control. However, using the percentage changes between real and sham conditions reported by Kaski et al. [28], we postulate that a sample size of 18 participants will be enough to detect a medium effect size between the real and sham a-tDCS groups (Cohen’s *d* = 0.5) with an estimated SD change of ± 25 for the retropulsion pull test. The LOS and SOT measures were not used by Kaski et al. [28]; therefore, the retropulsion pull test was selected because it contains a balance perturbation similar to that of the SOT. Therefore, to accommodate for a 20% dropout rate, we aim to recruit 8 participants per group (24 participants in total).

### Statistical analysis

Statistical analyses will be conducted using Stata version 11 statistical software (StataCorp, College Station, TX, USA). A generalised linear mixed model with factors for time (0, 6, 12 and 24 weeks), group (exergaming + sham a-tDCS vs exergaming + real a-tDCS vs control) and group-by-time interaction will be used to determine the effect that different direct current stimulation interventions have on brain excitability and balance. Potential factors such as the participant’s age and time since diagnosis will be used as covariates for analyses within our models. Per-protocol analysis will also be performed by including all participants who are at least 85% compliant with the exercise (as measured by the number of exercise sessions attended). Post hoc analyses using the Bonferroni correction will be applied as appropriate. Pearson’s product-moment correlation coefficient will be used to measure the level of correlation between change scores of the functional tests and brain excitability and activation. Significance will be set at *P* < 0.05.

When possible, we will try to obtain all endpoint measures from any dropouts in the final analysis. For participants who are lost to follow-up, missing data will be handled using the multiple imputation method [63]. Because the multiple imputation method assumes only that data are missing at random [64], we will additionally perform sensitivity analyses to evaluate the potential effect of non-random attrition [65]. Sensitivity analyses will involve testing a range of plausible scenarios in outcomes for participants who were lost during follow-up [66].

## Discussion

Falls among people with PD incurs a considerable cost associated with hospitalisation and injury management or treatment. An economic report published in 2014 established that health system costs due to accidental falls represent an additional 22% of the total health cost of PD [67]. This translates to an additional estimated annual cost of $1487AUD per person with PD [67]. Exergaming is a mode of exercise that is engaging, adds diversity to a rehabilitation programme, can be performed at home and may be effective at improving functional capacity and reducing risk of falls in people with PD. However, by combining exergaming with a-tDCS (a primer for adaptive neuroplasticity), greater and longer-term improvements in motor function may be achieved. To date, there is limited evidence for the effectiveness of combined exergaming and a-tDCS to improve balance and lower limb function in people with PD. As such, we aim to use a double-blind, randomised controlled study protocol to explore the efficacy of concurrent a-tDCS and exergaming for improving static and dynamic balance over a period of 12 weeks. In addition, our secondary objectives of using TMS and fNIRS to measure neurophysiological changes in the M1 and DLPFC will potentially provide a mechanistic understanding of any functional changes observed. Last, few studies have addressed the consolidation of long-term motor skill retention following exercise and a-tDCS in people with PD, and therefore we aim to explore any residual improvements in balance, lower limb and/or neurophysiological function after a 12-week follow-up period.

In our study, exergames are used to deliver cognitive and static and dynamic balance exercises for people with PD. The Jintronix software used in our study is capable of using an inexpensive, commercially available motion capture camera to deliver a vast selection of rehabilitation games from a personal computer or laptop. Additionally, the use of a-tDCS to augment adaptive neuroplasticity may lead to greater and longer-lasting improvements in balance and lower limb function in people with PD. Thus, we hypothesise that (1) balance will improve for the exergaming groups compared with controls and (2) the addition of real a-tDCS will provide additive improvements. Novel home care therapies that are easily administered are likely to maintain exercise enjoyment and facilitate long-term exercise adherence. Therefore, if this trial is successful, this intervention will potentially allow for a high degree of scalability into a community- or home-based setting, where exergames can be implemented either as part of a rehabilitation programme in health-care facilities (i.e., rehabilitation hospitals, age care facilities and nursing homes) or as an adjunctive therapy that can be performed at home in conjunction with standard therapy. Our study also has the potential to have wider implications for reducing total health-care costs (i.e., hospitalisation, injury management and assistive care costs) associated with falls for people with PD.

### Trial status

Eighteen participants have completed 24 weeks of training and are waiting to be tested at 3-month follow-up. No dropouts or adverse events have been reported.

## Additional file

Additional file 1: Standard Protocol Items: Recommendations for Interventional Trials (SPIRIT) 2013 checklist: recommended items to address in a clinical trial protocol and related documents. (DOC 122 kb)

## Abbreviations

1RM: One-repetition maximum
10MWT: 10-Metre Walk Test
4SST: Four Square Step Test
5TSTST: Five Times Sit to Stand Test
AE: Adverse event
a-tDCS: Anodal transcranial direct current stimulation
COP: Centre of pressure
CS: Conditioning stimulus
DLPFC: Dorsolateral pre-frontal cortex
fMRI: Functional magnetic resonance imaging
fNIRS: Functional near-infrared spectroscopy
HHb: Deoxygenated haemoglobin
ICC: Intraclass correlation coefficient
ISI: Interstimulus interval
LICI: Long-interval intracortical inhibition
LOS: Limits of Stability Test
M1: Primary motor cortex
mBest: Mini Balance Evaluation Systems Test
MCI: Mild cognitive impairment
MEP: Motor-evoked potential
O_2_Hb: Oxygenated haemoglobin
PD: Parkinson’s disease
QoL: Quality of life
RMT: Resting motor threshold
sEMG: Surface electromyography
SICI: Short-interval intracortical inhibition
SOT: Sensory Organization Test
SPIRIT: Standard Protocol Items: Recommendations for Interventional Trials
TA: Tibialis anterior
tDCS: Transcranial direct current stimulation
TMS: Transcranial magnetic stimulation
TS: Test stimulus
UPDRS: Unified Parkinson’s Disease Rating Scale
